# Human fetal tissue is critical for biomedical research

**DOI:** 10.1016/j.stemcr.2023.10.008

**Published:** 2023-11-16

**Authors:** Justin Brumbaugh, Brian A. Aguado, Tamra Lysaght, Lawrence S.B. Goldstein

**Affiliations:** 1 Department of Molecular, Cellular, and Developmental Biology, University of Colorado Boulder, Boulder, CO, USA; 2 University of Colorado Cancer Center, Anschutz Medical Campus, Aurora, CO, USA; 3 Charles C. Gates Center for Regenerative Medicine, University of Colorado Anschutz Medical Campus, Aurora, CO, USA; 4 Department of Bioengineering, University of California, San Diego, La Jolla, CA, USA; 5 Sanford Consortium for Regenerative Medicine, La Jolla, CA, USA; 6 Centre for Biomedical Ethics, Yong Loo Lin School of Medicine, National University of Singapore, Singapore, Singapore; 7 Department of Cellular and Molecular Medicine, University of California, San Diego, La Jolla, CA, USA; 8 Department of Neurosciences, University of California, San Diego, La Jolla, CA, USA

## Abstract

Human fetal tissue and cells derived from fetal tissue are crucial for biomedical research. Fetal tissues and cells are used to study both normal development and developmental disorders. They are broadly applied in vaccine development and production. Further, research using cells from fetal tissue is instrumental for studying many infectious diseases, including a broad range of viruses. These widespread applications underscore the value of fetal tissue research and reflect an important point: cells derived from fetal tissues have capabilities that cells from other sources do not. In many cases, increased functionality of cells derived from fetal tissues arises from increased proliferative capacity, ability to survive in culture, and developmental potential that is attenuated in adult tissues. This review highlights important, representative applications of fetal tissue for science and medicine.

## Teaser

Human fetal tissue and cells derived from human fetal tissue are important for many areas of research. This review outlines primary applications for fetal tissue as (1) a gold standard comparator for model systems, (2) a resource to study development, (3) a tool to study infectious diseases, and (4) a system to develop vaccines, with representative examples for each area.

## Introduction

**Figure 1:**
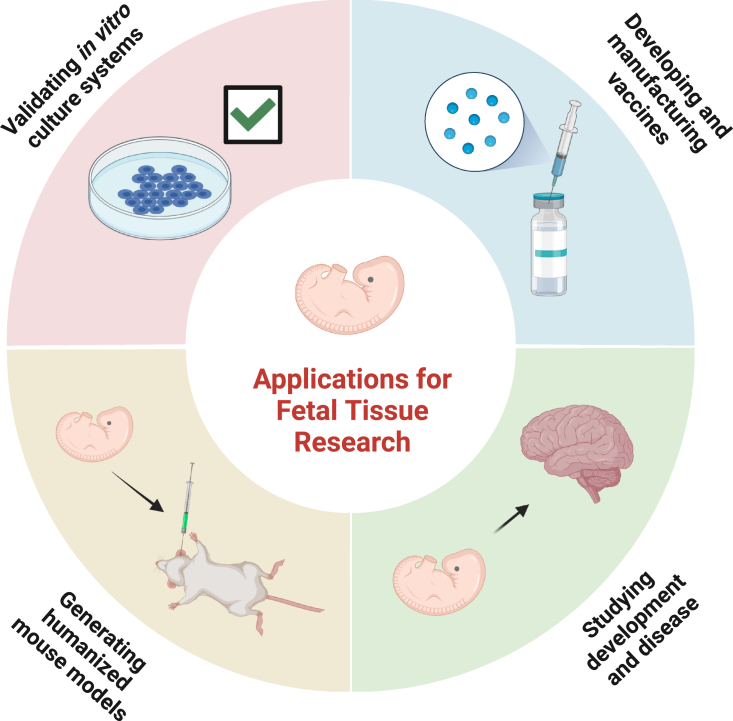
Human fetal tissue is used for important research and medical purposes

Human fetal tissue (HFT), defined by the NIH as tissue or cells obtained from a dead human embryo or fetus after a spontaneous or induced abortion, is an important resource for biomedical research (Boonstra, 2016; Wadman, 2015). Studies on HFT provide information on development and disease that cannot be accurately recapitulated in animal models or cell culture systems. Moreover, cells derived from HFT often have expanded proliferative or differentiation capacity compared with cells derived from adult tissue (Institute of Medicine (US) Conference Committee on Fetal Research and Applications, 1994). Based on these distinguishing features, HFT and cell lines derived from HFT have been used since the 1930s for many applications in research and medicine (Table 1; Figure 1) (Boonstra, 2016). These applications have ultimately saved millions of lives, particularly through vaccine development and production. Moreover, HFT has played a fundamental role in validating and optimizing protocols to generate specialized cells or tissues from stem cells. In this way, HFT has facilitated the use of stem cells for basic science and regenerative medicine, which underscores the continuing value of HFT research.

**Table 1:** Applications for human fetal tissue

Application	Examples of specific uses	Why fetal tissue is ideally suited for this application	Representative references
Validating *in vitro* culture systems	Confirming and optimizing stem cell-derived models of retinal pigment epithelium and kidney	Stem cell and animal models do not always recapitulate mature, human tissues. Fetal tissue provides access to effectively all cell types as a standard.	Wadman (2015); Ferrer et al. (2014); Lindström et al., 2018; O’Brien et al. (2016)
Developing and manufacturing vaccines	Developing and manufacturing vaccines for polio, measles, mumps, rubella, varicella, herpes zoster, adenovirus, rabies, and hepatitis A	Certain cells derived from fetal tissue survive and proliferate in culture more readily than adult cells and can support large amounts of viral production for making many vaccines.	Olshansky et al. (2017); Wadman (2015)
Studying development and disease	Studying diabetes, placental development and infertility, emerging diseases (Zika and SARS-CoV-2), neurodegenerative disorders (Alzheimer’s disease)	Fetal tissue provides an authentic *in vivo* context to study complex developmental processes and disease.	Ravassard et al. (2011); Scharfmann et al. (2014); van der Torren et al. (2016); Horii et al. (2021); Li et al. (2013); Karvas et al. (2022); Soncin et al. (2018); Israel et al. (2012); Cataldo et al. (2000); Mlakar et al. (2016)
Generating humanized mouse models	Used to study viral infections (HIV), allergies, and autoimmune disorders	Cells from fetal tissue more accurately recapitulate human systems and can be used to study fetal systems in the *in vivo* context of a small animal.	Fujiwara (2018); Wadman (2015); Adoro et al. (2015); Murooka et al. (2012); Balazs et al. (2014); Lu et al. (2016); Olesen et al. (2016)

Despite its substantial contribution to medicine and science, with many demonstrable benefits, significant public debate persists surrounding the use of HFT in biomedical research (Wadman, 2015). HFT is sourced from human fetuses postmortem and is typically procured following either electively or spontaneously terminated pregnancies (https://crsreports.congress.gov/product/pdf/R/R44129/10; https://www.govinfo.gov/content/pkg/GAOREPORTS-GAO-01-65R/pdf/GAOREPORTS-GAO-01-65R.pdf); however, tissue obtained from spontaneous terminations (i.e., miscarriages) is less likely to be recoverable in a viable state and more likely to carry genetic defects, limiting its use for research (Spach et al., 2021; Strong, 1991). It is important to note that while some applications require whole, primary tissue that must be collected before each experiment, most research uses cell lines derived from fetal tissues that can be passaged extensively and stored for later use. In fact, many of the most widely used fetal cell lines, including the HEK293, WI-38, and MRC-5 cells lines, were derived in the 1970s and 1980s but are still in use throughout the world today.

Setting aside the regulatory and ideological complexities of these debates, the goal of this review is to provide a resource for the public, governmental, and scientific communities that documents important applications of HFT in biomedical research. While the specific uses for fetal tissue are too numerous to thoroughly catalog in a single review, we have identified four primary research areas that rely on fetal tissue and provide examples for each. We focus on fetal tissue as (1) the gold standard for comparison to model systems, (2) a fundamental resource to study development, (3) a crucial tool to study infectious disease, and (4) an indispensable system for vaccine development and manufacture. Finally, we examine alternative materials to HFT, considering the scientific progress that must occur before such materials can adequately replace HFT. Together, the information presented here illustrates why HFT is a unique resource that is important for improving our understanding of disease and our efforts to improve human health.

## Applications

### Fetal tissue is the gold standard

By guiding pluripotent stem cells to assume particular identities, it is possible to make specific cell types for regenerative therapies, disease modeling, or drug testing. Therefore, directing cell fate *in vitro* to engineer functional, developmentally suitable cells is a fundamental goal in stem cell biology and often a crucial step toward developing new therapies. In many cases, however, cells generated from the directed differentiation of pluripotent stem cells do not fully recapitulate their *in vivo* counterparts. For example, neurons derived from pluripotent stem cells sometimes exhibit aberrant electrophysiology and gene expression patterns that are characteristic of immature cells (Belinsky et al., 2011; Kayama et al., 2019; Patterson et al., 2011). Consequently, it is crucial to validate and optimize differentiation protocols by comparing the properties of cells produced from pluripotent stem cells with cells derived from fetal tissue. HFT provides a potential source of essentially all cell types and therefore has served as an important standard (Wadman, 2015).

There are many examples that demonstrate the importance of HFT as a comparative standard, which is highlighted by studies of eye development and disease (Wadman, 2015). Retinal pigment epithelium (RPE) is a single-layer, pigmented epithelium that covers the back of the eye. Its deterioration is associated with macular degeneration and is a leading cause of blindness in the developed world. Over the past 20 years, methods for generating RPE have improved significantly (Kashani et al., 2018), aided in many cases by fetal tissue research (Zhou et al., 2015). Fetal tissue served as a direct comparator for RPE derived from human pluripotent stem cells (Ferrer et al., 2014) and cell lines derived from fetal tissue were important for establishing culture conditions needed to maintain and study RPE *in vitro* (Maminishkis et al., 2006; Wadman, 2015). As a result, stem cell-derived RPE therapies are undergoing clinical trials and so far, appear both safe and potentially effective (da Cruz et al., 2018; Kashani et al., 2018; Lawley et al., 2015; Mandai et al., 2017; Schwartz et al., 2015; Seiler and Aramant, 2012). It is worth noting that although some treatments are available to slow certain kinds of macular degeneration, no current therapy is available to restore RPE and visual function. It is possible that, in the future, transplantation therapies can be advanced through HFT research to restore patients’ vision by RPE replacement.

Fetal tissue and cell lines derived from fetal tissue have also been a valuable benchmark for assessing model systems used to study the kidney. A substantial part of our understanding of kidney development and disease is based on rodent models. While these models are relevant to the human kidney, comparative studies of the human fetal kidney highlight limitations in rodent models (Lindström et al., 2018a). For example, there are obvious differences in cell types and development between mouse and human kidneys. Specifically, gene regulatory programs differ between mouse and human kidney progenitors (O’Brien et al., 2016), which could impact progenitor cell function and differentiation. Further, the slower speed of human kidney development, compared with rodents, permits greater resolution of developmental events, revealing differentiation mechanisms not reported in rodent studies (Lindström et al., 2018b). Identifying and accounting for these differences is only possible through comparison with cells from HFT. Given the limitations of rodent-centered studies for improving human health, the NIH has supported several efforts to generate, curate, validate, and disseminate high-quality data from human fetal kidneys (National Institutes of Health (NIH), 2022). Collectively, these efforts provide an important resource for studying kidney disease, which affects over 800 million people worldwide and costs more than $70 billion annually in the United States for individuals over 66 years old (National Institutes of Health (NIH), 2022).

Overall, fetal tissue has provided a point of comparison for numerous *in vitro*-derived cells and tissue model systems. Notably, some of these systems now serve as important and accessible alternatives to fetal tissue research; however, validation and optimization of each system remains an essential step in establishing new models that will ultimately direct therapeutic efforts.

### Fetal tissue is a tractable model to study development and disease

Many adult diseases and disorders result from early developmental defects (Wadman, 2015). Understanding the origins of disease and establishing relevant treatments requires research on the developmental processes that take place in both normal and abnormal tissue. Fetal tissue has been indispensable in this regard because it offers a source of material from an authentic developmental context. There are extremely broad applications for studying development and disease using HFT. Outlined below are aspects of HFT-based research on a subset of diseases, including diabetes, placental development, neurological disorders, and emerging diseases.

#### Diabetes

Type 1 diabetes affects millions of people worldwide. In affected individuals, insulin-producing pancreatic beta cells are lost, causing inadequate insulin production and the inability to properly control blood glucose levels. Over time, unbalanced blood glucose leads to serious disease complications including cardiovascular disease, neuropathy, kidney damage, eye damage, and other associated pathologies. To regulate blood glucose levels, many patients with diabetes are forced to administer insulin, costing $327 billion per year in the United States (Yang et al., 2018). Consequently, identifying durable treatments for diabetes is an important therapeutic goal. Replacing lost beta cells through transplantation can help to control disease symptoms (Paty et al., 2013); however, deriving mature, functional islet cells from stem cells *in vitro* is extremely challenging (Hrvatin et al., 2014) and insulin-producing cells from the adult pancreas do not grow in the laboratory long-term. As a result, access to insulin-producing cells from HFT has been essential to understand how they develop. This has led to improved protocols for generating insulin-producing cells from pluripotent stem cells that can be transplanted into patients with diabetes. Pluripotent stem cell-derived beta cells are currently being tested in promising clinical trials (e.g., NCT04786262; NCT03163511).

Pancreatic cells from HFT have also been used to study how these cells respond to glucose levels in the blood and how this response can be modulated in diabetic individuals. To address this need, researchers used tissue (fetal age 7–11 weeks of gestation) to successfully generate insulin-producing cell lines that respond more realistically to blood glucose levels (Ravassard et al., 2011; Scharfmann et al., 2014). Cells derived from fetal tissue have also aided efforts to understand why, in type 1 diabetes, insulin-secreting cells are depleted by autoimmunity (van der Torren et al., 2016). Further, studying cells from fetal tissue has highlighted important differences between human and animal insulin-secreting cells, clarifying discrepant findings in other species and improving the chances for development of successful therapies (Brozzi et al., 2015).

#### Placentation

Fetal tissue research has been crucial for understanding placental development as well. Approximately one in five women will experience infertility, and defective placentation causes common pregnancy disorders such as pre-eclampsia, fetal growth restriction, and pre-term birth. The placenta develops from trophoblast stem cells (TSCs), which arise within embryos following fertilization. Identification of the molecular pathways that lead to proper placental formation has been limited by the paucity of tools available to study this process. Recently, however, researchers succeeded in generating trophoblast stem cell lines and organoids from fetal tissue (Haider et al., 2018; Okae et al., 2018; Turco et al., 2018). TSC lines and trophoblast organoids provide an important window into placental development but can only be derived from early first-trimester placental tissues (<10 weeks gestational age). These lines also serve as an important resource for drug testing and disease modeling during placentation. Based on this work, induced pluripotent stem cell (iPSC)-derived models of human TSCs are now available (Horii et al., 2016, 2019, 2021; Li et al., 2013), as are organoids (Karvas et al., 2022). Though more work is needed to optimize and validate these approaches, this example illustrates the importance of fetal tissue research for establishing alternative model systems. Additional placental cell types, including cytotrophoblast cells have also been derived from first-trimester placenta. Using these lines, researchers demonstrated that signaling through hypoxia-inducible factor regulates the generation of invasive extravillous trophoblast cells (Wakeland et al., 2017). Extravillous trophoblast cells establish the maternal-fetal interface and their disruption results in pre-eclampsia and fetal growth restriction. More recently, researchers profiled extravillous trophoblast cells isolated from first-trimester and term placenta at both the genomic and transcriptomic levels. The studies demonstrated that extravillous trophoblast cells become polyploid as they mature, limiting their survival in the uterus following pregnancy (Morey et al., 2021). Through these studies, researchers have been able to define the molecular mechanisms that control proper placentation and that are disrupted in conditions that can lead to developmental defects. Use of human tissue for this work is essential, as placental development is vastly different between species. Indeed, many genes that are expressed in the early gestation human trophoblast are not observed in mice and some placental cell types are quite distinct in humans and mice (Soncin et al., 2018).

#### Alzheimer’s disease

Many diseases of the brain affect both children and adults, ranging from autism to psychiatric disorders to neurodegenerative disorders. Accumulating evidence suggests that many of these disorders arise from aberrant gene expression and other phenotypic changes during fetal development. One specific example is Alzheimer’s disease, which is a disease that manifests during adulthood. Fetal tissue research has been important in validating the identity of neural cells in Alzheimer’s disease models generated from human iPSCs (Israel et al., 2012). An intriguing discovery is that early neural defects that precede Alzheimer’s disease can be found in individuals affected by Down syndrome, and Alzheimer’s disease consistently manifests in individuals with Down syndrome after age 40 (Cataldo et al., 2000). Specifically, the pathological enlargement of neuronal early endosomes, an early marker of Alzheimer’s disease, was observed in cells from the brain of a fetus affected by Down syndrome, well before other Alzheimer’s disease pathologies appear (Cataldo et al., 2000). Studies using Down syndrome fibroblasts at fetal and postnatal ages subsequently revealed that endosome dysfunction was dependent on the causative Alzheimer’s disease gene, APP, and more specifically on elevated levels of its direct cleavage product (Jiang et al., 2010). Additional work on endocytosis in stem cell-based neuronal models with Alzheimer’s disease mutations subsequently supported the appearance of early phenotypes (Mertens et al., 2021). These findings have been instrumental in guiding therapeutic interventions targeting endosome dysfunction in clinical trials.

#### Emerging diseases

Given the emergence of new pathogens such as Zika virus and SARS-CoV-2, access to HFT is also crucial in studying how such pathogens affect pregnancy. Zika virus infection, which is usually benign in adults, can have devastating effects on the developing human fetus. In otherwise healthy pregnant individuals, the Zika virus crosses the placental barrier, where it can infect the developing brain and cause microcephaly and other related malformations (Mlakar et al., 2016). Animal and cell culture models are helpful but insufficient for studying the disease, partly because they lack essential cell types involved in infection and viral spread. The use of fetal tissue has provided crucial insight into how Zika viruses cross the placenta to infect brain cells (Mlakar et al., 2016; Nowakowski et al., 2016; Retallack et al., 2016a; Tabata et al., 2016). Work from these and other studies has informed screening efforts to identify Food and Drug Administration-approved drugs to protect fetuses from Zika virus (Retallack et al., 2016b). The ability to identify the developmental origins of disease is crucial to understanding early pathology. A range of other pathogens from viruses and bacteria to parasites, such as Toxoplasma, can also infect the fetus and lead to severe diseases in children. Understanding how some pathogens cross the placental barrier, whereas others do not, is critical to finding ways to protect fetuses from infection. This research greatly benefits from the use of cells isolated from human placentas and/or fetuses (Megli and Coyne, 2022).

Recently human TSCs from fetal tissue were used to evaluate SARS-CoV-2 infection in early gestation, demonstrating that while the virus does not infect the stem cells themselves, it readily infects both extravillous trophoblast and syncytiotrophoblast cells derived from TSCs. These findings suggest different routes of viral entry and perhaps modes of injury when infection occurs in early gestation (Kallol et al., 2021). Consistent with this, scientists have observed distinct effects of SARS-CoV-2 on the placenta of patients infected during their first trimester, with correspondingly higher rates of placental injury compared with uninfected individuals (Lokken et al., 2021). Given the importance of placental function in supplying oxygen and nutrients to the developing fetus, placental injury increases the risk of pregnancy complications including miscarriage. Fetal tissue has also been used to determine which neural cell types are susceptible to SARS-CoV-2 infection (Andrews et al., 2022). This work revealed that astrocytes are readily infected by SARS-CoV-2, while neurons are largely refractory to infection. An important finding from the study was the identification of CD147 and DPP4 as receptors involved in astrocyte infection by SARS-CoV-2, which provides a more complete mechanistic understanding of the process (Andrews et al., 2022). The authors note that specific cell vulnerabilities may help to explain recurring neuropsychiatric symptoms experienced by many individuals following SARS-CoV-2 infection, including dizziness, seizures, and cognitive problems (Andrews et al., 2022).

### Fetal tissue is irreplaceable for generating humanized mouse models

Studying human development and disease often requires *in vivo* approaches, but some studies are difficult or impossible to conduct entirely within humans. Non-human, animal models have been indispensable in this regard; however, many differences exist between human and animal systems that influence cell and tissue physiology (Fujiwara, 2018; Wadman, 2015). One way to address this limitation while retaining the *in vivo* context is by engrafting human cells into mice, thus generating “humanized mouse models.” A variety of tissues have been studied this way, including liver, skin, neural tissue, and the hematopoietic and immune system (Fujiwara, 2018). Researchers have used HFT, including cells from bone marrow, liver, spleen, thymus, and lymph node (and in various combinations) to make humanized mice (Wadman, 2015); however, it is also possible to generate humanized mice from non-fetal tissue or perinatal tissues, like bone marrow or umbilical cord blood (Allen et al., 2019; McCune and Weissman, 2019). While these systems are useful for certain applications, they cannot fully recapitulate development processes modeled by cells derived from fetal tissue (McCune and Weissman, 2019). For example, humanized mice derived from adult cells cannot be used to study the maturation of fetal T cells, as the transplanted cells have already matured beyond this developmental time point (Bunis et al., 2021). Thus, while some alternative systems are available, humanized mice created using HFT remain an important and valuable research tool. For information on the types of humanized mouse models, uses, and cell sources, we refer the reader to comprehensive reviews (Allen et al., 2019; Garcia and Freitas, 2012; Ito et al., 2012).

Many viruses relevant to human health infect only human cells; therefore, humanized mice offer an important opportunity to study host-pathogen relationships in a small animal context. The use of humanized mice to study numerous viral infections, allergies, and autoimmune disorders is reviewed extensively elsewhere (Fujiwara, 2018). Here, we detail studies involving human immunodeficiency virus (HIV). HIV attacks the body’s immune system, destroying CD4^+^ T cells, macrophages, and dendritic cells that are critical for fighting infection. If left untreated, HIV can lead to acquired immunodeficiency syndrome (AIDS), with an average survival of just 3 years when untreated (Centers for Disease Control and Prevention [CDC] https://www.cdc.gov/hiv/basics/whatishiv.html). In the United States, more than 1.2 million people are living with HIV, and it is a leading cause of death in developing countries (CDC https://www.cdc.gov/hiv/basics/whatishiv.html). While the virus can be controlled with access to good medical care, the cost of treatment remains substantial.

A primary challenge in AIDS research is establishing tractable models to study HIV infection and disease progression because many animal species that are commonly used in biomedical research, including rodents, are not infected by HIV. Moreover, non-human primates can be infected by simian immunodeficiency virus, not HIV (Wadman, 2015). However, the hematopoietic and immune systems of mice can be humanized by transplanting human hematopoietic stem cells into immunocompromised mice, where they give rise to human blood and immune cells, including T cells that can be infected by HIV. This provides an *in vivo* model that has been used to study HIV infection and HIV therapies. Indeed, studies using humanized mice have provided insight into HIV biology that would not have been possible in other systems. For example, research from mice engrafted with human fetal bone marrow, liver, and thymus (i.e., “BLT mice”) identified key host responses that mediate viral replication and disease progression (Adoro et al., 2015). Other studies in humanized mice revealed that HIV-infected T cells are highly motile and form long, membrane tethers that enhance the spread of HIV through affected lymph nodes (Murooka et al., 2012). In other cases, humanized mice have been used test treatments for HIV based on neutralizing antibodies (VRC07, 3BNC117) (Balazs et al., 2014; Klein et al., 2012; Lu et al., 2016) and to study how combinatorial antiretroviral therapy (Viread, emtricitabine, raltegravir) reduces viral transmission (Lu et al., 2016). This work highlights the importance of humanized mice as a fundamental tool in studying HIV and AIDS.

### Vaccine development and production relies on cells derived from HFT

The emergence of modern vaccines has improved human health, saving millions of lives annually. Immunization against polio, measles, mumps, rubella, varicella, herpes zoster, adenovirus, rabies, and hepatitis A was estimated to prevent a combined 4.5 billion cases of disease and 10.5 million deaths globally between 1960 and 2015 (Olshansky and Hayflick, 2017). Producing reliable, consistent vaccines requires a standardized cell line to generate large amounts of viral material for immunizations. In the 1960s, researchers at the Wistar Institute and the Medical Research Council Laboratory developed stable cell lines from HFT, namely WI-38 and MRC-5, that were capable of substantial expansion in culture and produced large amounts of target virus (Wadman, 2015). Due to these features, cell lines derived from fetal tissue are a crucial tool for developing vaccines against many pathogens (CDC, 2021; Institute of Medicine (US) Conference Committee on Fetal Research and Applications, 1994; Wadman, 2015; 2017). In some cases, fetal cell lines are still used to generate vaccines, though we note that no fetal cells are included in the final immunization. Below are some examples.

#### Polio

Poliovirus is highly contagious and, in unvaccinated individuals, may lead to paralysis and death. In the late 1940s, researchers discovered that poliovirus expanded readily in cell lines derived from HFT, a breakthrough that allowed large-scale production of material for vaccines and dramatically improved human health in the United States (College of Physicians of Philadelphia). In the 1940s, before the vaccine was widely available, the CDC reported 35,000 cases of paralytic poliovirus (Centers for Disease Control and Prevention, 2022). Through federal vaccination programs, incidence of the disease dropped to less than 100 in the 1950s. At present, polio has been nearly eradicated from the Western hemisphere and is rare worldwide (Centers for Disease Control and Prevention, 2022).

#### Rubella

Though rubella virus causes only mild symptoms in most children and adults, infection during pregnancy can cause miscarriage or congenital rubella syndrome in affected children. Access to cell lines derived from HFT was essential for both the isolation and attenuation of the rubella virus during vaccine development, leading to the virtual elimination of rubella in the United States (College of Physicians of Philadelphia). The vaccine is still made using the human fetal cell line, WI-38, as this vaccine is safer and more effective than rubella vaccines developed using non-human cells.

#### Varicella (chickenpox)

Chickenpox infections are typically benign but can have serious complications resulting in pneumonia or encephalitis. Prior to vaccine development, between 50 and 100 children died from chickenpox annually in the United States alone (College of Physicians of Philadelphia). Additionally, when contracted during gestation, chickenpox infection can cause death or serious birth defects for the child. Notably, the chickenpox virus does not expand robustly in non-human cells, so the vaccine must be produced using the MRC-5 or WI-38 human fetal lines (College of Physicians of Philadelphia).

#### Hepatitis A

Hepatitis A is caused by a small RNA hepatovirus that infects the liver. Like vaccines for chickenpox and rubella, the hepatitis A vaccine, Havrix, is generated by passaging the virus in HFT lung cell lines, prior to harvest and attenuation. Havrix is currently generated using MRC-5 cells derived from fetal tissue (CDC, 2021).

#### SARS-CoV-2

The emergence and spread of SARS-CoV-2 virus necessitated the rapid development of safe and effective vaccines (Watson et al., 2022). Cell lines derived from fetal tissue were fundamental to these efforts. For example, the AstraZeneca ChAdOX1 nCoV-19 and the Johnson & Johnson (Jansen) Ad26.COV2.S vaccines are manufactured in cell lines derived from fetal tissue (HEK293 and PER.C6, respectively) (Zimmerman, 2021). The mRNA vaccines generated by Pfizer-BioNTech and Moderna are not produced in cells derived from fetal tissue but the HEK293 fetal cell line was used during preclinical testing of the vaccines (Zimmerman, 2021). Fetal cell lines are frequently used for this purpose because they offer a consistent standard against which safety and efficacy are evaluated. Overall, cell lines derived from fetal tissue were instrumental in the development of SARS-CoV-2 vaccines, which researchers estimate prevented 14.4 million deaths worldwide over a 1-year period between 2020 and 2021 (Watson et al., 2022).

Other vaccines manufactured in cells from HFT include adenovirus, shingles, Haemophilus influenza type b (Hib), and rabies. These vaccines continue to protect people across the world from serious infectious diseases, saving billions in healthcare costs and preventing lifelong disability and death.

## Ethical and regulatory considerations

A central goal in biomedical research is to generate knowledge that improves the understanding of biology and promotes human health. In assessing the value of research, well-established and widely accepted ethical principles guide researchers to consider the potential benefits and burdens of research, and to use materials that are procured ethically from legal sources (American Medical Association, 2017; Holm, 2002; National Institutes of Health Office of Intramural Research, 2021; The Public Health and Welfare, 2010). Researchers are generally not involved in the procurement of fetal tissues from donors but obtain them from third-party suppliers that are responsible for collecting, storing, and distributing tissues according to prevailing laws and regulations (https://crsreports.congress.gov/product/pdf/R/R44129/10; https://www.govinfo.gov/content/pkg/GAOREPORTS-GAO-01-65R/pdf/GAOREPORTS-GAO-01-65R.pdf). Given that fetal tissue is primarily sourced from elective pregnancy terminations, the ethics of HFT research are sometimes conflated with political and ideological debates around abortion. Those debates are complex and warrant a separate, comprehensive review.

Legal and regulatory frameworks that apply to HFT have an important role in supporting or restricting research (MacDuffie et al., 2021; McCune and Weissman, 2019) and therefore scientific advances. Funding, for example, is dramatically influenced by political ideology and may be subject to rapid change, depending on the ruling party. Abrupt fluctuations in funding have devastating consequences for biomedical research that rely upon these resources to complete long-term studies (Servick, 2021). An additional layer of complexity is that policy can vary significantly between, and sometimes within, jurisdictions. For example, in the United States alone, applicable regulations will depend on the source of funding, the type of research (e.g., preclinical research, clinical trials, etc.), the state, the institution where the research is performed, and the jurisdiction where the tissue was procured (American Medical Association, 2017; National Institutes of Health Office of Intramural Research, 2021; The Public Health and Welfare, 2010). Different rules may also apply to cells lines that are processed and distributed commercially, as compared with research with whole fetal tissues. In Europe, HFT research is regulated under the directives, regulations, and guidelines of the European Union, which are ratified and enacted in national regulatory frameworks of individual countries (de Wert et al., 2002). Throughout Asia, each country where elective terminations are legal has their own regulatory frameworks, of which some are more formalized than others (https://www.bioethics-singapore.gov.sg/publications/reports/bac-ethics-guidelines-2021) (Yui et al., 2023).

Generally speaking, though, regulations aim to avoid any influence on the decision to terminate a pregnancy by ensuring that the potential donor’s decision to undergo termination is separated from and made prior to obtaining informed consent to donate any postmortem fetal tissues to research. Additionally, international guidelines emphasize that the donation of fetal tissues be voluntary and, in accordance with this principle, they prohibit payments or valuable consideration for the donation of fetal tissues for research (https://www.isscr.org/guidelines) (Lovell-Badge et al., 2021). It is hoped that any countries or regions that are developing regulations on fetal tissue research would consider the values and benefits of the research and appropriate ethical measures described above.

## Alternatives to HFT in research

**Figure 2:**
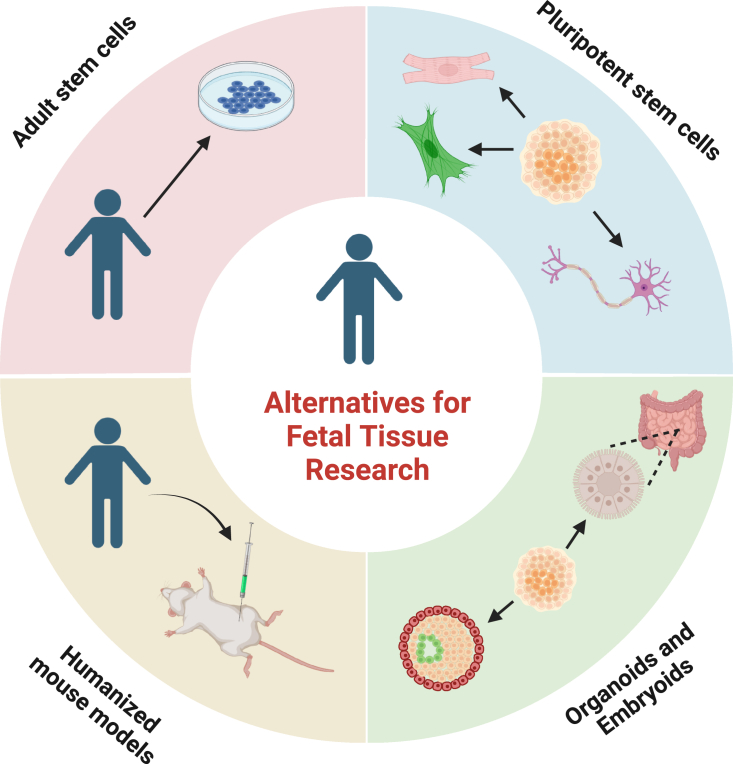
Alternatives to human fetal tissue for research and medicine

While HFT research has led to many scientific advances, it is important to consider alternatives to HFT. Current and emerging alternatives to HFT include the use of tissues and adult stem cells derived from postnatal individuals, pluripotent stem cells, organoids, and humanized mouse models using adult cells (Figure 2). We emphasize, however, that in many cases research has not yet established that alternative cell sources possess the necessary properties to fully replace HFT.

### Adult stem cells

Adult stem cells (ASCs) have been used for a multitude of biomedical research endeavors and have saved the lives of millions worldwide (Prentice, 2019). ASCs are found in many tissues, including perinatal tissues like umbilical cord blood, and adult tissues like blood, bone marrow, skin, and muscle. Various types of ASCs have been harnessed for regenerative therapies and have become the standard for treating disorders of the blood-forming and immune systems. In some applications, ASCs can be used to obtain cell types that model differentiation or tissue homeostasis. For example, mesenchymal stem cells are an adult stem cell type capable of generating bone, fat, and cartilage cells (Pittenger et al., 2019). Mesenchymal cells are therefore a valuable system to study differentiation for these lineages; however, most ASCs are specialized to their tissue of origin and can only differentiate into a limited range of cell types relative to fetal stem cells (Clevers and Watt, 2018; Zakrzewski et al., 2019). Moreover, adult cells are not suitable for some applications, such as vaccine production, as they are less likely to survive and proliferate in culture as compared with cells derived from fetal tissue (https://www.ncbi.nlm.nih.gov/books/NBK231997/).

### Induced pluripotent stem cells

Human iPSCs are genetically reprogrammed from adult somatic cells such as skin cells or leukocytes and can differentiate into any cell type in the adult body. Several studies have used iPS cells in various differentiation protocols to generate cells from a wide range of tissues and organs, including nerve cells, cardiac cells, liver cells, and blood cells. As such, iPSCs can be used to generate any cell type in the body from the donor, mitigating immune responses when the cells are transplanted autologously back into the same patient. Additionally, iPSCs have wide utility for drug screening, *in vitro* disease modeling, and regenerative medicine applications. As noted above, cells differentiated from iPSCs can have properties that differ from normal cells *in vivo*, requiring validation in comparison with cells from fetal or adult tissues. Moreover, not all conditions needed for certain developmental processes can be recapitulated *in vitro*. In particular, the fetal thymus is necessary for programming T cell receptors and self-recognition, which currently cannot be achieved *in vitro* with pluripotent stem cells. Thus, although iPSCs are a valuable alternative in many cases, HFT remains an important research tool for some tissues.

### Organoids

Research that uses fetal tissue offers the important opportunity to study homeostasis and disease states directly in the context of complex tissues and organs. In many cases, it is difficult to recapitulate these complex structures *in vitro*; however, organoids have emerged as an approach to model certain tissues in culture. Organoids are multicellular, self-organizing cell aggregates that are grown *in vitro* and that recapitulate the three-dimensional architecture found in some tissues. Organoids can be derived from pluripotent stem cells, fetal cells, or adult tissues (Clevers, 2016). Their multicellular, three-dimensional structures can be used to simulate disease processes and are useful for capturing physiological processes *in vitro* (Takebe and Wells, 2019). For example, organoids have been used to determine how viral infections (including SARS-CoV-2) may impact some tissues (Andrews et al., 2022; Mallapaty, 2020). Although organoids derived from adult cells circumvent some limitations of HFT, organoids still lack globally standardized protocols, some cells cannot be grown as organoids, and organoids commonly have properties that differ from normal cells *in vivo* (Kim et al., 2020).

### Stem cell-based embryo models

Emerging research suggests that human pluripotent stem cells can be used to generate three-dimensional cell aggregates in culture, known as stem cell-based embryo models (e.g., blastoids) (Clark et al., 2021). Previously, donated human embryos were cultured *in vitro* to model pre- and peri-implantation development (Shahbazi et al., 2016). As a potential alternative, models derived from pluripotent stem cells can be used to recapitulate some aspects of early embryonic development (Kagawa et al., 2022; Liu et al., 2021; Weatherbee et al., 2023; Yanagida et al., 2021; Yu et al., 2021). Although stem cell-based embryo models represent a significant step forward in studying embryogenesis*,* it is not yet clear whether all aspects of development, including gene expression and epigenetic profiles, are accurately recapitulated in these systems. Further work is necessary to determine whether these approaches will serve as a true alternative for fetal tissue. As such, fetal tissues remain a critical source of cells to understand human development and serve as a crucial point of comparison for benchmarking stem cell-based embryo models. Moreover, generating stem cell-based embryo-like structures is likely raise to other ethical considerations that have been reviewed in detail elsewhere (Clark et al., 2021; Rivron et al., 2023).

## Conclusion

The aim of sharing the information compiled here is to highlight the important, representative applications of HFT in biomedical research. Collectively, research using HFT has saved millions of lives, decreased healthcare costs, and substantially improved human health. Moving forward, HFT will remain a crucial resource for medicine and basic science. It is imperative that policymakers and the public recognize the vast medical applications and advances that have, and will, come from the use of HFT in biomedical research. While alternative models may reduce reliance on this material, it is not possible to fully leverage the alternatives without further validation using HFT. Thus, for researchers to develop new therapies and to understand human development and disease progression, it is essential that policymakers and the general public consider the regulation and funding of HFT from an informed perspective.
